# Bovine HDL and Dual Domain HDL-Mimetic Peptides Inhibit Tumor Development in Mice

**DOI:** 10.17303/jcrto.2020.8.101

**Published:** 2020-01-17

**Authors:** Feng Su, Anantharamaiah GM, Mayakonda N. Palgunachari, C. Roger White, Holly Stessman, Yanyuan Wu, Jay Vadgama, Richard Pietras, Dorothy Nguyen, Srinivasa T. Reddy, Robin Farias-Eisner

**Affiliations:** 1 Department of Obstetrics and Gynecology, David Geffen School of Medicine, the University of California at Los Angeles, Los Angeles, CA 90095, USA; 2 Department of Medicine, the University of Alabama at Birmingham, Birmingham, AL 35294, USA; 3 Department of Pharmacology, Creighton University Medical School, Omaha, NE 68178, USA; 4 Division of Cancer Research and Training, Charles R. Drew University of Medicine and Science, Los Angeles, CA 90059, USA; 5 Department of Internal Medicine, Charles Drew University, Los Angeles, CA 90059, USA; 6 Jonsson Comprehensive Cancer Center, the University of California at Los Angeles, Los Angeles, CA 90095, USA; 7 Department of Medicine, Division of Cardiology, David Geffen School of Medicine, the University of California at Los Angeles, Los Angeles, CA 90095, USA; 8 Department of Molecular and Medical Pharmacology, David Geffen School of Medicine, the University of California at Los Angeles, Los Angeles, CA 90095, USA; 9 Department of Obstetrics, Gynecology, School of Medicine, Creighton University, Omaha, NE 68178, USA; 10 Hereditary Cancer Center, School of Medicine, Creighton University, Omaha, NE 68178, USA

**Keywords:** HDL, Mimetic Peptides, Ovarian Cancer, Colon Cancer, LPA, Cancer Therapeutics

## Abstract

A growing body of literature supports the role of apolipoproteins present in HDL in the treatment of pro-inflammatory diseases including cancer. We examined whether bovine HDL (bHDL) and three dual-domain peptides, namely AEM-28 and its analog AEM-28–2, and HM-10/10, affect tumor growth and development in mouse models of ovarian and colon cancer. We demonstrate that bHDL inhibits mouse colorectal cancer cell line CT26-mediated lung tumor development, and mouse ovarian cancer cell line ID8-mediated tumor burden. We also demonstrate that, although to different degrees, dual-domain peptides inhibit cell viability of mouse and human ovarian and colon cancer cell lines, but not that of normal human colonic epithelial cells or NIH3T3 mouse fibroblasts. Dual-domain peptides administered subcutaneously or in a chow diet decrease CT26 cell-mediated tumor burden, tumor growth, and tumor dissemination in BALB/c mice. Plasma levels of lysophosphatidic acid (LPA) are significantly reduced in mice that received bHDL and the dual-domain peptides, suggesting that reduction by effecting accumulation and/or synthesis of pro-inflammatory lipids may be one of the mechanisms for the inhibition of tumor development by bHDL and the dual-domain peptides. Our studies suggest that therapeutics based on apolipoproteins present in HDL may be novel agents for the treatment of epithelial adenocarcinomas of the ovary and colon.

## Introduction

When considering the cohort of patients with gynecologic malignancy, the deadliest of these cancers are the high-grade epithelial adenocarcinoma of Mullerian cell origin (e.g. ovarian, fallopian tube, primary peritoneal papillary serous adenocarcinoma) and collectively represents the 5th most frequent cause of cancer-related deaths in women overall. In spite of its extremely low incidence (an annual incidence of 11.6 cases/100,000 women per year) [1], the WHO reports a significant global impact, approximately 225,500 new cases of epithelial ovarian cancer (EOC) will be diagnosed worldwide annually, with approximately 140,200 (62%) of these patients, succumbing to their disease [1]. Colon cancer (CC), similarly is one of the most devastating diseases and represents the second deadliest cancer (second only to lung cancer), and the third most common cancer diagnosed, in the United States [2]. The American Cancer Society estimates the occurrence of 101,420 new cases of colon cancer in the United States for 2019 [2]. Although the survival rates for many solid tumors have improved over the past 50 years, survival data from numerous countries reported that the 5-year overall survival of EOC patients has remained virtually unchanged over the past 40 years, in large part because of the ultimate development of disease that is chemotherapy resistant [3–5]. With very few early non-specific symptoms, EOC often eludes the clinician, and the majority of patients will have a disease that is already disseminated widely throughout the peritoneal cavity at the time of initial diagnosis [4]. Moreover, although the initial response to frontline platinum-based chemotherapy is excellent, following optimal cytoreductive surgery, recurrence is inevitable in over 50% of patients, with decreasing time intervals between disease recurrences, and the eventual emergence of chemotherapy-resistant and refractory disease is inescapable, at the present time [5]. A similar clinical picture is also common in patients with colon cancer diagnosed under the age of 50 [6]. Hence, the development of novel forms of targeted therapy is necessary to positively impact patient survival and reduce the morbidity and mortality burden associated with these devastating cancers [7–10].

Recently, several targeted therapies (e.g. PARP inhibitors, immunotherapy, and antiangiogenic agents) have emerged as a potential treatment for chemotherapy-resistant EOC, albeit with little impact on long-term survival [7–9]. Based on new research, most new CC cases are universally screened for DNA mismatch repair and microsatellite status as well as RAS and BRAF mutational testing [10]. Although the screening has aided prognosis and predicted efficacy, there has not been any positive impact on overall survival in patients with refractory colon cancer [10]. Research in the past decade from several laboratories including our own identified lipid metabolism as a key component of cancer growth and development [11]. Elucidation of the molecular mechanisms that regulate lipid metabolism and cancer is an important area of current research.

High-density lipoprotein (HDL) is an important mediator of lipid homeostasis. HDL and HDL-associated molecules exert a number of protective effects including strong anti-inflammatory, antioxidant, anti-microbial functions and an activator of innate immunity in multiple cell types and animal models [12–13]. HDL mimetic peptides (18 to 28 amino acid residues in length) have shown efficacy in a number of animal models of disease and demonstrate properties that make them attractive as potentially effective therapeutic agents [14].

Apolipoprotein A-I (ApoA-I), apolipoprotein E (apoE), and apolipoprotein J (apoJ) are important components of HDL and play roles in HDL’s reverse cholesterol transport and anti-inflammatory properties. The amphipathic α-helix is a common structural motif that enables apoA-I, apoJ and apoE to achieve these functionalities. The concentration of HDL was found to be inversely associated with the risk of ovarian and colon cancer [15]. Compared to human plasma, bovine plasma is almost exclusively HDL (Human: HDL 80mg/dl, LDL 136mg/dl; Cattle: HDL 118mg/dl, LDL 8mg/dl) [16]. Thus, we examined whether bHDL exerts anticancer effects *in vitro* and *in vivo*. Our previous studies demonstrated that apoA-I, apoJ and apoE mimetic peptides inhibit tumor development in mouse models of ovarian and colon cancer [17–21], and play an important anti-atherogenic role in facilitating the clearance of LDL from the circulation [22]. The receptor-binding domain of apoE (140–151 residues with the sequence L-R-K-L-R-K-R-L-L-R, hE) was covalently linked to either an apoA-I mimetic peptide called 18A or an apoJ sequence (L-V-G-R-Q-L-E-E-F-L, corresponding to amino acids 113 to 122 in apoJ), to obtain dual-domain peptides AEM-28 and HM-10/10, respectively.

Therefore, in this study, we examined whether bHDL and the dual-domain HDL mimetic peptides, AEM-28, AEM-28–2, and HM-10/10, exert anticancer effects *in vitro* and *in vivo*.

## Materials and Methods

### Mice

The Animal Research Committee at the University of California at Los Angeles approved all mouse protocols. 9-week-old female C57BL/6J mice and 6-week-old female BALB/c mice were purchased from The Jackson Laboratory.

### Bovine HDL (bHDL)

Bovine HDL (bHDL) used in this study was isolated from bovin plasma and purified via ultracentrifugation, sequential high-speed centrifugal flotation in KBr (1.063–1.21g/cc) to homogeneity. This was determined via agarose gel electrophoresis and SDS-PAGE next to human HDL. Protein determinations were performed by the Lowry method [23]. bHDL preparations were membrane filtered and contained 0.15M NaCl and 0.3mM EDTA at pH 7.4.

### Peptides

The dual-domain peptide apoE mimetic peptide −28 (AEM-28) has the amino acid sequence

“L-R-K-L-R-K-R-L-L-R-D-W-L-K-A-F-Y-D-K-V-A-E-K-L-K-E-A-F” derived by covalently linking the heparin-binding domain 141–150 (L-R-K-L-R-K-R-L-L-R) of apoE and 18A (D-W-L-K-A-F-Y-D-K-V-A-E-K-L-K-E-A-F), a class A amphipathic helical peptide. The dual-domain peptide HM-10/10 (Ac-L-R-K-L-R-K-R-L-L-R- L-V-G-R-Q-L-E-E-F-L-NH_2_ contains hApoE[141–150] and an apoJ mimetic named G* peptide {Ac-L-V-G-R-Q-L-E-E-F-L-NH_2_ corresponding to amino acids 113 to 122 of apoJ (L- [113–122] apoJ)}. The peptide Ac-Aha-[R]hE-18A-NH_2_, called AEM-28–2 has the amino acid sequence Ac-Aha-L-R-R-L-R-R-R-L-L-R-D-W-L-K-A-F-Y-D-K-V-A-E-K-LK-E-A-F-NH_2_ and developed to increase potency and limit local irritation, the modifications were done to the original peptide Ac-hE18A-NH_2_. Lys residues in L-R-K-L-R-K-R-L-L-R were replaced by Arg since the replacement of Lys by Arg reduced cytotoxicity [24]. Ac-Aha- (Aha= α-aminohenoic acid) was added to the N-terminus instead of Ac- to enhance lipid binding. In this study, we also used the peptides L-4F and sc-4F as controls. The peptides were dissolved in H_2_O for the experiments.

### Cell-Culture Experiments

Mouse colorectal cancer cell line CT26, NIH3T3 cells, mouse ovarian cancer cell line ID8, human colon cancer cell line Caco-2, human normal colonic epithelial cells CCD 841 CoN, human ovarian cancer cell lines OV2008 and SKOV3, were cultured in their corresponding complete medium, which was replaced with serum-free medium before the experimental treatments. Following overnight incubation with serum-free medium, the cells were either treated with vehicle (H_2_O), or treated with 10μg/mL of peptide or bHDL at 10μg/ml or 100μg/ml. Cells were incubated for an additional 24 or 48 hours and assayed for viability using MTS assay kit (Promega) or apoptosis assay using the Annexin V-FITC Apoptosis Detection Kit (Invitrogen) according to the manufacturer’s protocols.

### ELISA Analysis

Interleukin (IL)-6 concentrations were measured in plasma by a competition ELISA kit from Invitrogen according to the manufacturer’s protocol.

### Tumor-Load Study

Six-week-old BALB/c female mice were given a 100ul subcutaneous injection of 1×10^6^ CT26 cells prepared as a single cell suspension in PBS, and treated with peptide at 10mg/kg subcutaneously daily for 15 days or treated with peptide at 10mg/kg by tail vein injection once a week for three weeks. The mice were sacrificed, and tumor weights were measured. The tumor volumes based on caliper measurements were calculated by using the formula V=1/2 (L × W^2^) or were measured by Vevo® 2100 system from VisualSonics.

Nine-week-old C57BL/6J mice were given an intraperitoneal injection containing 8 × 10^6^ ID8 cells in a total volume of 0.8 mL of DMEM (without supplements). Starting the same day, mice were treated with HM-10/10 at 100mg/kg or 4mg bHDL added to chow (Ralston Purina) diet/mouse/day, or only with standard chow diet as control. 10 weeks after the injection, the mice were sacrificed and tumor loads were assessed by counting the number of tumor nodules on the parietal peritoneal surfaces and the visceral peritoneal surfaces of the intestine, liver, kidney, and spleen.

### Pulmonary metastasis *in vivo*

BALB/c mice were intravenously injected with 2×10^4^ CT26 cells in 100μL of PBS via tail vein injection and the mice were treated with vehicle (0.15M NaCl and 0.3mM EDTA at pH 7.4) or bHDL (bHDL) at 10mg/kg/day/mouse administered subcutaneously for 3 weeks. After 3 weeks of treatment, the mice were sacrificed; lungs were harvested, weighed, and fixed with Bouin solution (Sigma). Tumor nodules on the lung surface were counted.

For the experiments using peptides, BALB/c mice were intravenously injected with 2×10^4^ CT26 cells in 100μL of PBS via tail vein and the mice were treated starting the same day with the peptide at 100mg/kg/day administered in a chow diet for 3 weeks, or fed with the regular chow diet. After 3 weeks of treatment, the mice were sacrificed; lungs were harvested, weighed, and fixed with Bouin solution (Sigma). Tumor nodules were counted, and lungs were fixed in formalin solution for sectioning. 1cm of jejunum was collected from each mouse to isolate lamina propria and another 1cm of jejunum was collected and fixed in formalin solution for sectioning.

### Flow Cytometry

Lamina propria isolated from the small intestines by using the lamina propria dissociation kit (Miltenyi Biotec Inc) according to the manufacturer’s protocol. Cells from lamina propria were labeled with fluorochrome-conjugated antibodies, anti-F4/80 (eBioscience) and anti-Ly6G (Miltenyi Biotec). FACS was performed using a BD LSR Fortessa X-20 machine in the Janis V. Giorgi Flow Cytometry Core Facility at UCLA. For analysis and computational compensation of the data, BD FACS Diva software was used. Ten thousand events of live cells were gated.

### Immunohistochemistry staining

Lung tissues and jejunum tissues were fixed and embedded with paraffin, sectioned at 5μm thickness. Sections were deparaffinized with xylene, rehydrated with 100%, 90%, 70%, and 50% ethanol, treated with proteinase K at 20μg/mL for 30 minutes, and treated with 3% H_2_O_2_ for 30 minutes at room temperature to inhibit endogenous peroxidase, blocked with 10% normal serum and 4% bovine serum albumin prepared in PBS for 3 hours, and then incubated with 1:50 anti-mouse F4/80 antibody, 1:1500 anti-Ly6G antibody overnight at 4°C. The sections were incubated with corresponding biotinylated secondary antibody for 1 hour, followed by incubation with Vectastain ABC Elite reagents.

### Statistical Analyses

The data are shown as means ± SD for each group. Statistical analyses were performed by the Student’s t-test. The difference between groups was established with Bonferroni’s post-hoc test. A *P* value of less than 0.05 was considered statistically significant.

## Results

### bHDL therapy inhibits CT26-mediated lung tumor development

**Figure. 1:**
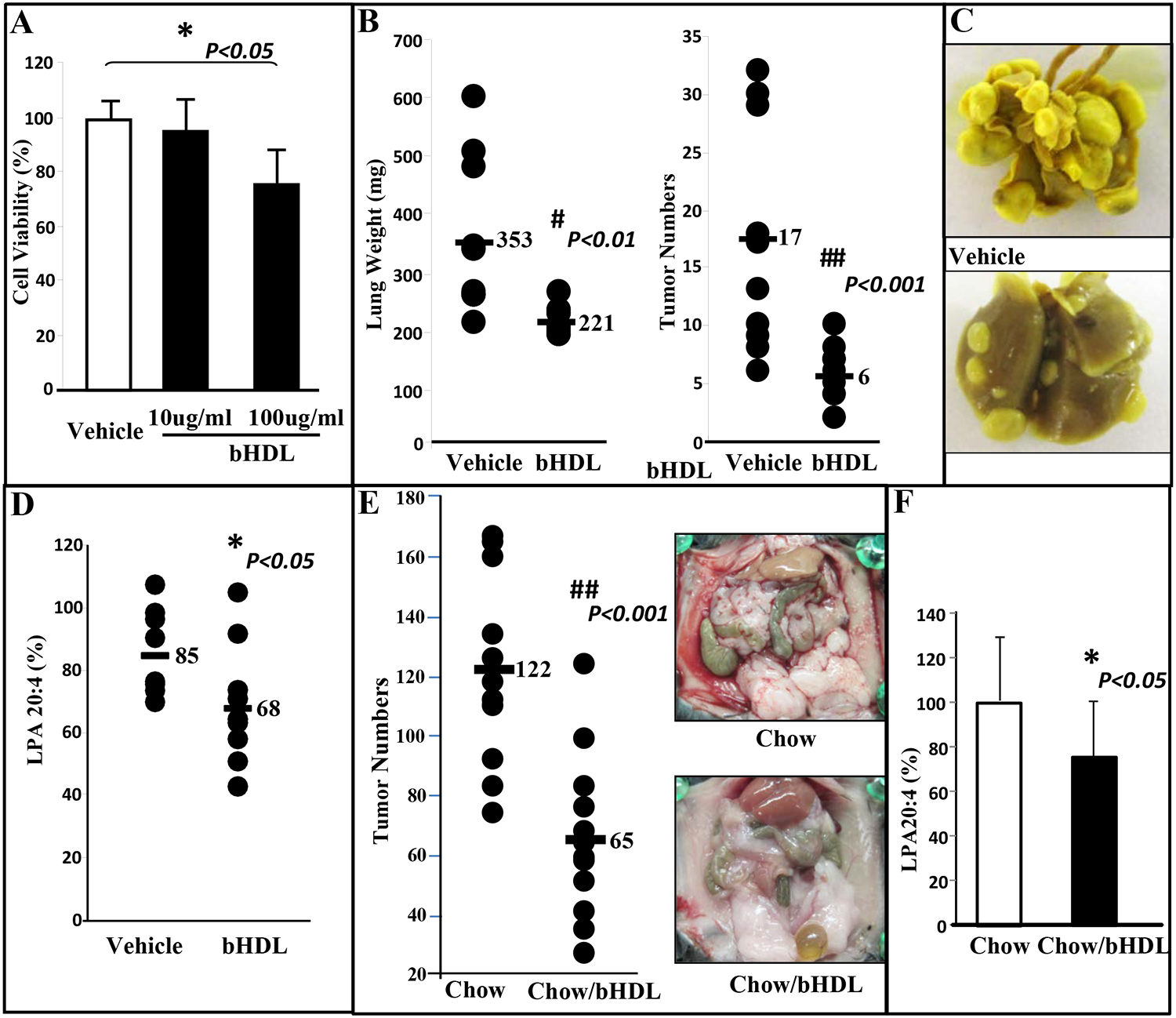
CT26 cell-mediated lung tumors in BALB/c mice and ID8 cell-mediated ovarian cancer burden in C57BL/6J mice are significantly decreased in mice administered bHDL. (**A**) CT26 cells were cultured as described in Materials and Methods and incubated with either vehicle (control) or bHDL at a concentration of 10ug/ml or 100ug.ml. CT26 cells were assayed for viability using MTS assay. Data are represented as the mean ± SD of the percent of control cells. All experiments were conducted in triplicate and each assay was carried out in quadruplicates. (**B**-**D**) Lung tumors were established in BALB/c mice (n=11 per group, 6 weeks of age) as described in Materials and Methods. Mice were sacrificed 3 weeks after CT26 cells were administered by tail vein injection. Lungs were harvested and weighed. Lung tumors were counted. (**B**) Left panel: lung weights of mice that were administered vehicle alone or bHDL (10mg/kg/day) subcutaneously (*p*< 0.01); Right panel: the number of tumors counted on the lung surface from the 2 groups of mice (*p*<0.001). (**C**) Representative tumors from the two groups of mice showing tumor nodules on their lung surface. (**D**) Plasma LPA 20:4 levels were measured from (B) as described under Materials and Methods. (**E**-**F**) Wild-type C57BL/6J mice (*n* = 11 per group, 8 weeks of age) were injected with ID8 cells by intraperitoneal injection (8 × 10^6^ cells per mouse) and tumor burden was analyzed after 10 weeks treated with a regular chow or a chow with bHDL. (**E**) Left panel: The total number of tumors nodules for each mouse was counted in each group; Right panel: representative images of mice from the two groups showing the tumor nodules on the peritoneal membranes. (**F**) Plasma LPA 20:4 levels in the two groups of mice from (E).

CT26 cell line has been widely used as a syngeneic tumor model to study therapeutic applications for colon cancer in mouse models. We first examined whether bHDL inhibits the growth of CT26 cells. Cell viability was approximately 30% lower in CT26 cells treated with bHDL (100μg/mL) when compared to no treatment controls (Figure 1A). We next examined the effect of bHDL *in vivo*. Pulmonary tumor development following CT26 cell injection was significantly decreased in mice treated with bHDL at 10mg/kg/day/mouse administered subcutaneously for 3 weeks (lung weights were 353 vs. 221 mg, *P*<0.01; tumor numbers were 17 vs. 6, *P*<0.001; Figure 1B). Representative photographs of lung tumors from 2 groups are shown in Figure 1C. LPA has been identified as an important mediator of tumor development, progression, and metastases in humans. In the experiment shown in Figure 1B, plasma LPA 20:4 levels were significantly reduced in mice that received bHDL compared with their corresponding control mice, *P*<0.05 (Figure 1D).

### Tumor burden following ID8 cell injection is significantly decreased in mice that received bHDL therapy in a chow diet

Immunocompetent mice develop into ovarian cancer when injected with ID8 cells (mouse ovarian cancer cell line). C57BL/6J mice were injected with ID8 cells by intraperitoneal injection (8 × 10^6^ cells per mouse; *n* = 11 per group). Mice received a regular chow diet or chow diet containing bHDL at 4mg/mouse/day. Tumor burden was analyzed after 10 weeks. Tumor load (average number of tumor nodules on liver, kidney, spleen, diaphragm, and intestines) was markedly greater in control C57BL/6J mice when compared with C57BL/6J mice treated with bHDL (122 vs. 65, *P*< 0.0001) (Figure 1E left panel). Representative photographs of tumor load from 2 groups are shown in Figure 1E right panel. Plasma LPA 20:4 levels were significantly reduced in mice that received bHDL compared with the control mice, *P*<0.05 (Figure1F). Our results suggested that bHDL administered either orally or SQ inhibit the development and progression of ovarian and colon tumors in mice.

### Tumor development following CT26 cell injection is significantly decreased in mice treated with AEM-28 administered subcutaneously.

**Figure. 2:**
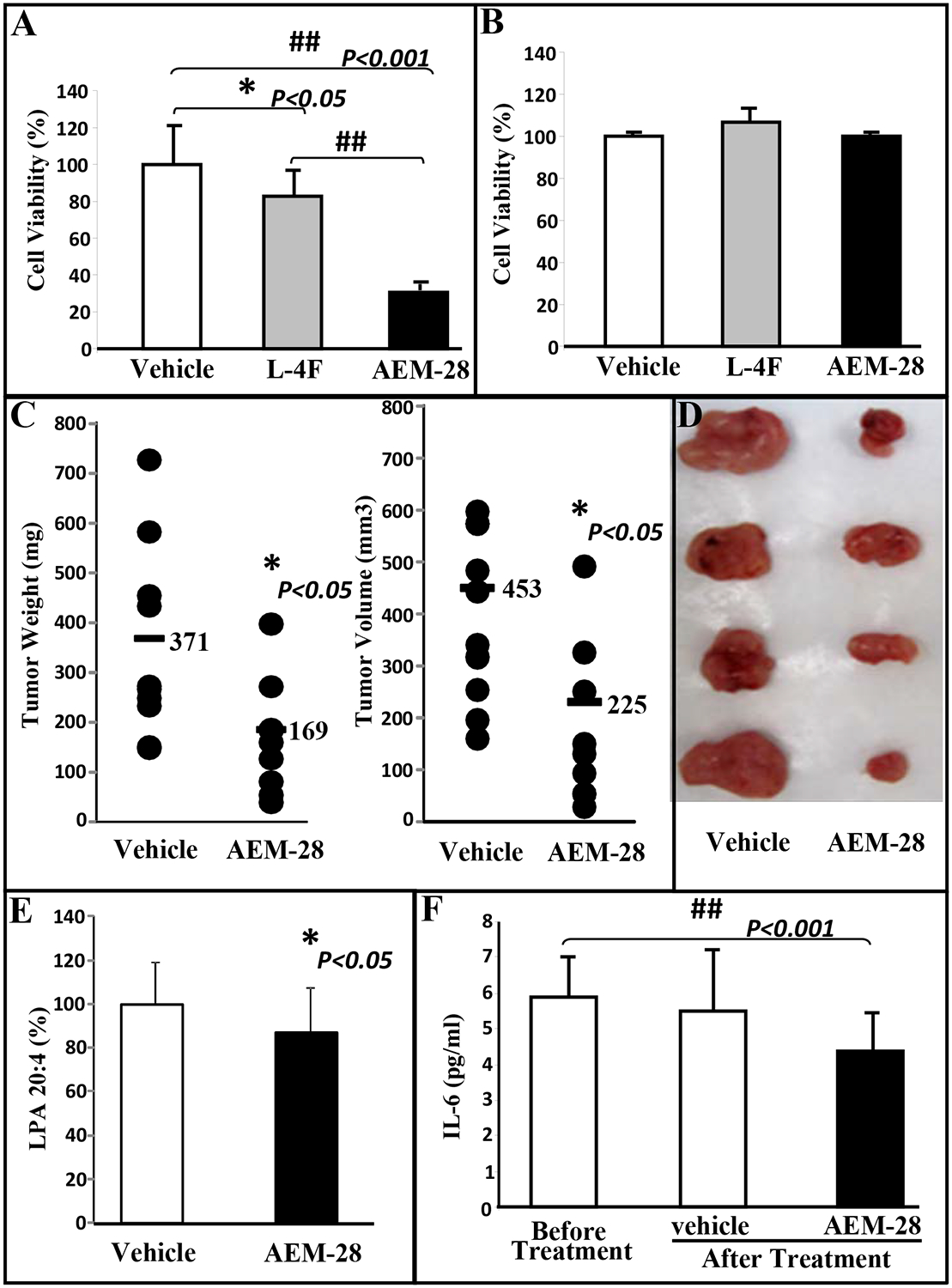
AEM-28 reduces the cell viability of CT26 cells and CT26 cell-mediated flank tumor growth in BALB/c mice. CT26 and NIH3T3 cells were cultured as described in Materials and Methods and incubated with either vehicle or 10μg/ml of either L-4F or AEM-28. (**A**) CT26 cell viability as measured by MTS assay is reduced in cells treated with L-4F and AEM-28. (**B**) AEM-28 and L-4F have no effect on cell viability of NIH 3T3 cells. All experiments were conducted in triplicate and each assay was carried out in quadruplicates. (**C**-**E**) Flank tumors were established in BALB/c mice as described in Materials and Methods. Mice were sacrificed 15 days after CT26 cell injections and with or without treatment of AEM-28 subcutaneously. Tumors were harvested, measured and weighed. (**C**) Left panel: tumor weights in mice receiving vehicle (n=11) or AEM-28(n=9); Right panel: tumor volumes from the 2 groups of mice. (D) Representative photographs of flank tumors from the 2 groups. (**E**) Plasma LPA 20:4 levels were measured from the experiment (C) as described under Materials and Methods. (**G**) Plasma IL-6 levels were measured before and after treatment as described under Materials and Methods.

In a series of experiments, we next examined the anti-tumorigenic potential of dual-domain peptides that were derived from functional mimetics of important HDL proteins. Since covalent addition of 140–151 receptor binding region of apoE to 18A in Ac-hE18A-NH_2_ peptide reduced plasma cholesterol in dyslipidemic animal models analogous to apoE protein, Ac-hE18A-NH_2_ is referred to as apoE-mimetic (AEM) peptide with 28 residues, i.e. AEM-28. We first examined the effect of AEM-28 on CT26 cell viability. AEM-28 significantly inhibited the viability of CT26 cells as measured by MTS assay (Figure 2A), but not NIH 3T3 cells (Figure 2B). L-4F, an apoA-I mimetic peptide significantly reduced CT26 cell viability as shown by us previously [20], however, reduction of CT26 cell viability by AEM-28 treatment was significantly greater than that of CT26 cells treated with L-4F (Figure 2A).

We next tested the effect of AEM-28 *in vivo*. BALB/c mice were injected with 1×10^6^ CT26 cells subcutaneously in the flank and treated with AEM-28 (n=10) or vehicle (n=12) at 10mg/kg/day administered subcutaneously for 15 days. The mice were sacrificed, and the tumor weights and volumes were measured. The tumor weights and volumes in BALB/c mice treated with AEM-28 were significantly reduced compared with mice treated with vehicle [weight: 370.75 vs. 168.8 mg, *P*<0.05. (Figure 2C left panel); volume: 452.8 vs. 225.2 mm^3^, *P*<0.05 (Figure 2C right panel)]. Representative photographs of tumors from 2 groups are shown in Figure 2D. LPA 20:4 levels in plasma were significantly decreased in mice with AEM-28 treatment compared with the control group, *P*<0.05 (Figure 2E). IL-6 levels were significantly decreased in mice treated with AEM-28 compared with the control mice, *P*<0.001 (Figure 2F).

### AEM-28 and AEM-28–2 inhibit the viability of mouse and human cancer cells

Anantharamaiah GM et.al. modified AEM-28 to increase potency and limit local irritation that was observed locally at injection sites. The modifications were done to the original peptide Ac-hE18A-NH_2_ by replacing the Lys residues by Arg since it has been previously shown that replacement of Lys by Arg reduced cytotoxicity [24]. Moreover, acylating or adding a hydrophobic amino acid that does not have a chiral center, such as NH_2_-(CH2)5-COOH increases hydrophobicity and thus enhances LDL association. Anantharamaiah GM et.al. selected NH_2_-(CH2)5-COOH, alpha-aminohexanoic acid for this purpose to modify AEM-28 [24]. To distinguish from AEM-28, its modified version (as described under Materials and methods) is called AEM-28–2.

**Figure. 3:**
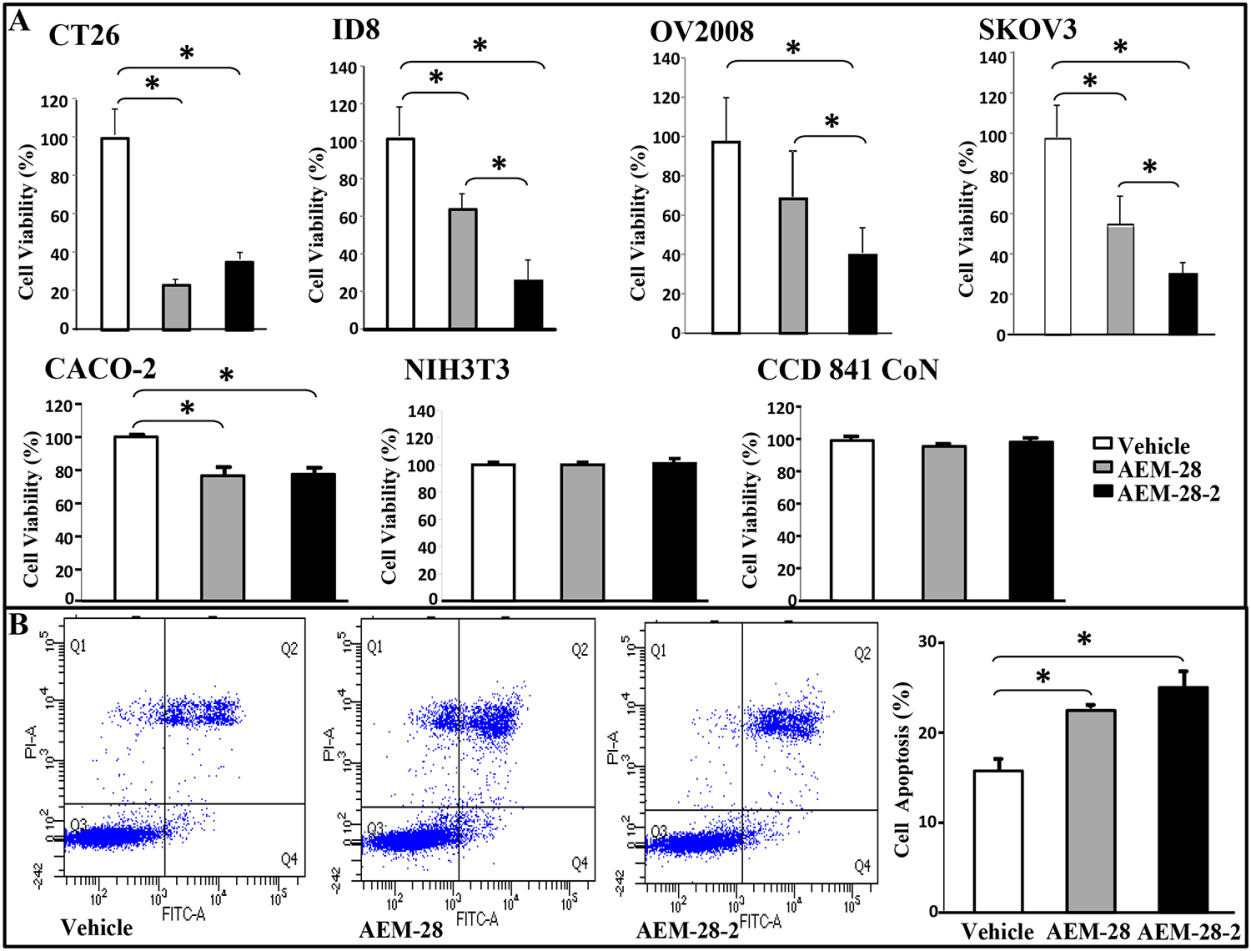
AEM-28 and AEM-28–2 reduce viability in human and mouse colonic and ovarian cancer cells but NIH3T3 and normal human colonic cells and induce apoptosis in CT26 cells *in vitro*. (**A**) All cells were cultured as described in Materials and Methods, and treated with either vehicle or AEM-28 or AEM-28–2 at a concentration of 10μg/ml. Cells were assayed for viability using the MTS assay. All experiments were performed in triplicate and each assay was carried out in quadruplicates. (**B**) The percentage of apoptotic cells of CT26 significantly increased with the peptide treatments when compared with control. The asterisk indicates *p*<0.016 with the Beonferroni correction.

The effects of AEM peptides on cell viability were determined using MTS assay *in vitro*. CT26 cell viability was reduced by over 70% (*P*<0.0001) with AEM-28 peptide (10 μg/ml) and reduced by 64% (*P*<0.0001) with AEM-28–2 (10 μg/ml), when compared to control cells (Figure 3A). Moreover, both AEM-28 and AEM-28–2 peptide significantly reduced the cell viability of ID8, OV2008, SKOV3, and CACO-2 cell lines (Figure 3A). AEM-28 and AEM-28–2 treatment did not affect cell viability in NIH3T3 cells and human normal colonic epithelial cell line CCD 841 CoN (Figure 3A). Moreover, AEM-28 and AEM-28–2 induced cell apoptosis measured by Annexin V-FITC/PI assay in CT26 cells when compared with control cells (Figure 3B). It should be noted that AEM-28–2 is better at significantly inhibiting tumor cell proliferation when compared to AEM-28.

### AEM-28 and AEM-28–2 peptides inhibit tumor development following flank injection of CT-26 cells in BALB/c mice.

**Figure. 4:**
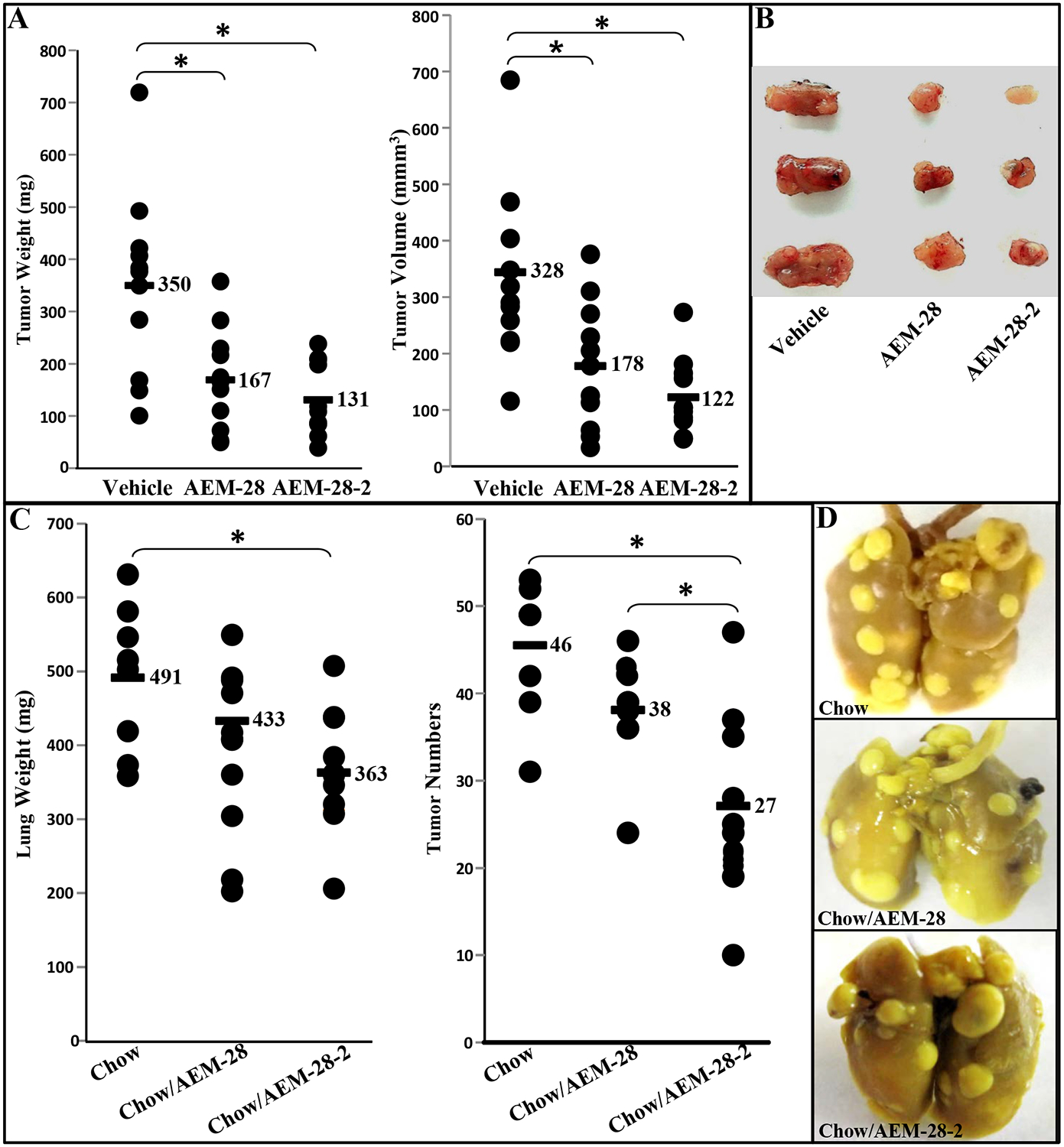
AEM-28 or AEM-28–2 significantly reduced CT26 cell–mediated flank tumors and lung tumors in BALB/c mice. (**A**-**B**) Flank tumors were established in BALB/c mice as described in Materials and Methods. Mice were sacrificed after 3 weeks with the treatment of vehicle or AEM-28 or AEM-28–2 administered by tail vein at 10mg/kg/week. Tumor weight and volume were measured. (A) Left panel: the data shown are tumor weights for mice receiving vehicle or AEM-28 or AEM-28–2 at 10mg/kg/week; Right panel: the data shown are tumor volumes. (B) Representative tumors are shown from three groups of mice. (**C**-**D**) Lung tumors were established in BALB/c mice as described in Materials and Methods. Mice were sacrificed 3 weeks after CT26 cells administered by tail vein injection. Lungs were harvested and weighed. Lung tumors were counted. (C) Left panel: the data shown are lung weights for mice receiving chow or AEM-28 or AEM-28–2 administered in a chow at 100mg/kg/day; Right panel: the data shown are the number of tumors counted on lung surface from the three groups of mice. (D) Representative tumors from the three groups of mice showing tumor nodules on the lung surface. Asterisk indicates *p*<0.016 with the Beonferroni correction.

We examined whether AEM-28 or AEM-28–2 treatment by tail vein injection affected the development of tumors in the flanks of BALB/c mice. Six-week-old BALB/c female mice were injected with 1×10^6^ CT26 cells subcutaneously in the flank. The mice were treated with either vehicle (*n*=11 per group) or AEM-28 or AEM-28–2 at 10mg/kg administered via tail vein weekly for three weeks. The flank tumor weights and tumor volumes were significantly larger in BALB/c mice treated with vehicle compared with mice treated with AEM-28 or with AEM-28–2 (350 vs. 167 vs. 131 mg, Figure 4A left panel; 328 vs. 178 vs. 122 mm^3^, Figure 4 A right panel). Representative photographs of flank tumors from 3 groups are shown in Figure 4B. We observed local irritation at the site of injections for AEM-28 but not for AEM-28–2. False-positive associations were ruled out by Bonferroni Correction for multiple testing. Once again, similar to tumor cell lines (Figure 3), AEM-28–2 is better at inhibiting tumor growth when compared to AEM-28.

### AEM-28 and AEM-28–2 peptides inhibit lung tumor development following the injection of CT-26 cells in BALB/c mice.

We next examined the effect of orally administered (in a chow diet at 100 mg/kg/day for 3 weeks) AEM-28 and AEM-28–2 on lung tumor formation in BALB/c mice injected with 2 × 10^4^ CT26 cells via tail vein. The lung weights and the tumor numbers counted on the lung surface in BALB/c mice treated with AEM-28 (*n*=9) and AEM-28–2 (n=12) were decreased (491 vs. 433 vs. 363 mg; 46 vs. 38 vs. 27) shown in Figure 4C. Representative photographs of lung tumors from 3 groups are shown in Figure 4D. In these studies, in contrast to AEM-28–2, AEM-28 did not significantly reduce lung tumor burden.

### Inflammatory cells in the intestinal and lung tissues are modulated by the dual-domain peptides

**Figure. 5:**
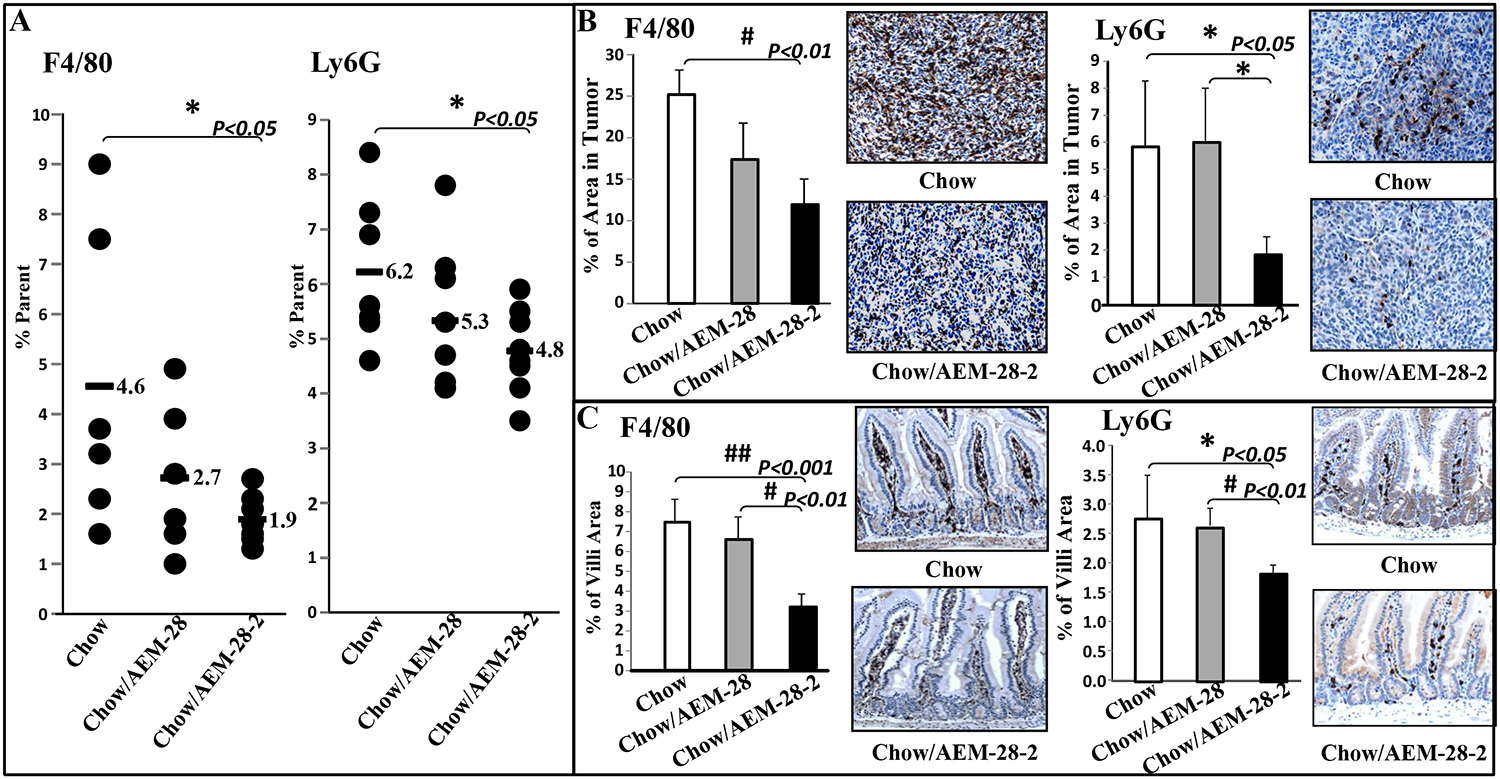
Flow cytometry analyses and macrophages expression of F4/80 and Ly6G in lung tumor tissue and jejunum tissue. (A) F4/80 and/or Ly6G are significantly reduced in mice after treatment with AEM-28–2 administered in a chow from FACS analyses. Cells were harvested from lamina propria described in Materials and Methods. The data have shown F4/80 and Ly6G in the percentage from lamina propria in Figure 5A. F4/80 and Ly6G immunostaining were performed on lung tumor tissue sections from the mice treated with different diets as described in Materials and Methods. (B) The quantification of F4/80 and Ly6G expression in tumor tissue on the lung surface. The brown stain represents F4/80 or Ly6G staining. F4/80 and Ly6G immunostaining were performed on jejunum tissue sections from the mice treated with a different diet. (C) The quantification of F4/80 and Ly6G expression in jejunum tissue. The brown stain represents F4/80 or Ly6G staining.

To better understand the mechanism behind the differential potencies between the two peptides and since the dual-domain peptides are effective through oral administration, we analyzed changes inflammatory cell populations in the lamina propria of mice treated with the two peptides. Flow cytometry analyses revealed a significant reduction in the percentage of F4/80 and Ly6G positive cells in the lamina propria harvested from mice treated with AEM-28–2 but not from AEM-28 (Figure 5A). Furthermore, IHC analysis revealed that both F4/80 and Ly6G expression in lung tumors (Figure 5B) and the jejunum tissues (Figure 5C), were significantly reduced in AEM-28–2 administered mice but not in AEM-28 treated mice.

### HM-10/10 peptide alters CT26 cell viability, inhibits LPA induced proliferation of CT26 cells *in vitro* and inhibits tumor development following flank injection of CT26 cells in BALB/c mice.

**Figure 6:**
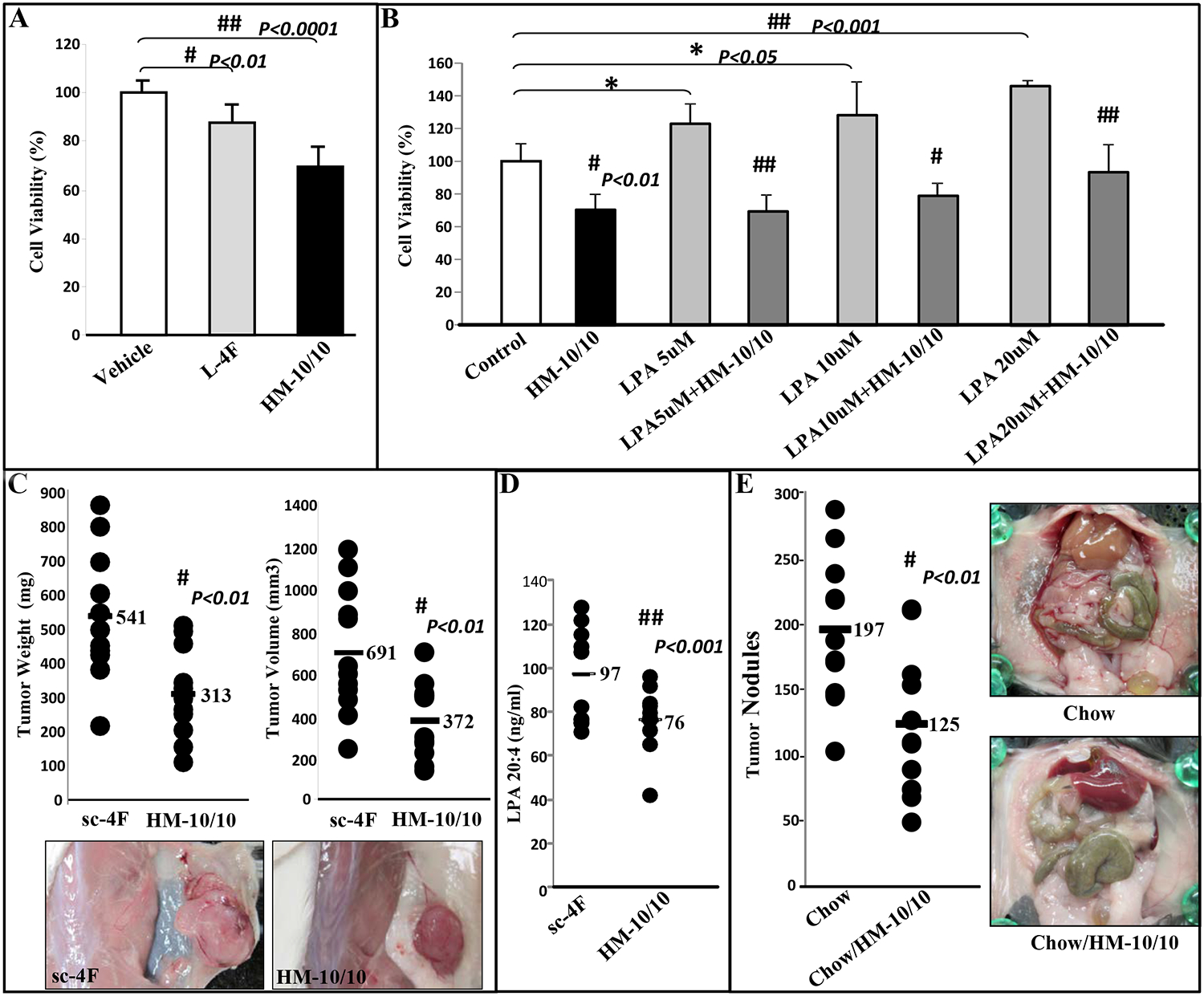
HM-10/10 alters CT26 cell viability and inhibits LPA induced proliferation of CT26 cells; CT26 cell-mediated flank tumors are significantly decreased in BALB/c mice treated with HM-10/10 by subcutaneously and ID8 cell-mediated ovarian cancer burden is significantly decreased in C57BL/6J mice receiving HM-10/10 in chow. (A) CT26 cells were cultured as described in Materials and Methods and incubated with either vehicle or L-4F or HM-10/10 at the concentration of 10μg/ml. CT26 cells were assayed for viability using MTS assay. (B) CT26 cells were cultured and incubated with either HM-10/10 (10μg/mL) or LPA at a concentration 5, 10, or 20 μM, or cells treated with both HM-10/10 and LPA for 48 hours. Data are represented as the mean ± SD of the percent of control cells. All experiments were conducted in triplicate and each assay was carried out in quadruplicates. (C-D) Flank tumors were established in BALB/c mice (n=12 per group). Six-week-old BALB/c female mice were given a 100μL subcutaneous injection of 1×10^6^ CT26 cells prepared as a single cell suspension in PBS, and the mice were treated with sc-4F or HM-10/10 at 10mg/kg administered subcutaneously daily for 15 days. The mice were sacrificed, and tumor volumes were measured by the Vevo® 2100 system from VisualSonics. (C) Left panel: the data shown are tumor weights for mice receiving sc-4F or HM-10/10 at 10mg/kg subcutaneously daily, *P*<0.01; Right panel: the data shown are tumor volumes from the two groups of mice, *P*<0.01. Bottom panel: representative photographs of flank tumors from the two groups are shown. (D) Plasma LPA 20:4 levels from the experiment of (C). (E) Wild-type C57BL/6J mice (*n* = 11 per group, 9 weeks of age) were injected with ID8 cells by intraperitoneal injection (8 × 10^6^ cells per mouse) and tumor burden was analyzed after 10 weeks. Left panel: The total number of tumors nodules for each mouse was counted in each group; Right panel: representative images of mice from the two groups showing the tumor nodules on the peritoneal membranes.

Based on our previously published data [20], we developed a second novel dual-domain peptide, HM-10/10 (described under materials and methods). We first measured cell viability by using MTS kit. Cell viability was reduced by more than 30% (*P*<0.00001) in CT26 cells treated with HM-10/10 (10μg/mL) when compared with control cells (Figure 6A). LPA (5–20 mM) significantly induced CT26 cell growth (Figure 6B) and HM-10/10 significantly inhibited LPA-induced cell viability at all doses tested (Figure 6B). We then examined the effect of HM-10/10 and negative control, sc-4F (D-W-F-A-K-D-Y-F-K-K-A-F-V-E-E-F-A-K), a peptide with the 4F sequence scrambled so that it does not form of a class A or class G amphipathic helix [20], on the development of tumors in the flanks of BALB/c mice. Six-week-old BALB/c female mice were injected with 1×10^6^ CT26 cells subcutaneously in the flank. The mice were treated with either sc-4F (n=12) or HM-10/10 (n=12) at 10 mg/kg administered subcutaneously daily for 15 days at a site distant from the site where the CT26 cells were injected. The mice were scanned, and the tumor weights and volumes were measured by Vevo® 2100 system from VisualSonics. The tumor weights and volumes in BALB/c mice treated with HM-10/10 were significantly smaller compared with mice treated with sc-4F [weights: 541 vs. 313 mg, *P*<0.01 (Figure 6C left panel); Volume: 691 vs. 372 mm^3^, *P*<0.01) (Figure 6C right panel)]. Representative photographs of flank tumors from 2 groups are shown in the bottom panel of Figure 6C. We also measured LPA levels in plasma. LPA levels (LPA 20:4; LPA 18:0 and LPA 18:1) were significantly decreased in mice with HM-10/10 treatment compared with control group. LPA 20:4 levels are shown in Figure 6D.

### Tumor burden following ID8 cell injection is significantly decreased in mice treated with HM-10/10 in chow.

C57BL/6J mice were injected with ID8 cells by intraperitoneal injection (8 × 10^6^ cells per mouse; n = 11 per group). Mice received a regular chow diet or the peptide HM-10/10 at 100mg/kg/day in a chow. After 9 weeks of treatment, tumor nodules on the liver, kidney, spleen, diaphragm, and intestines were counted. Tumor load was significantly decreased in C57BL/6J mice treated HM-10/10 in chow when compared with the mice received a regular chow (125 vs. 197, *P*< 0.01) (Figure. 6E left panel). Representative photographs of tumor load from 2 groups are also shown in Figure 6E right panel.

## Discussion

Complete clinical and biochemical responses are rare in recurrent chemotherapy-refractory EOC and CC. It is essential to discover novel effective therapies to greatly reduce the morbidity and mortality and to save the many lives lost to these devastating cancers. The studies in this paper are the first report describing the potent anticancer effects of bHDL, and the dual-domain peptides, AEM-28, AEM-28–2, and HM-10/10 in mouse models of EOC and CC. Remarkably, the dual-domain peptides markedly inhibit the viability of human chemotherapy-resistant ovarian cancer cell lines (e.g. OV2008 and SKOV3). Our data support the hypothesis that HDL and HDL-associated apolipoproteins function as novel agents for the treatment of chemotherapy-refractory, EOC and CC, cancers. Our data support a mechanism of action consistent with reduced LPA levels, which were significantly decreased in mice that received bHDL and the three dual-domain peptides, suggesting that LPA reduction may be a common mechanism for the inhibition of tumor development.

Several pro-inflammatory disease states including cancer, diabetes mellitus, systemic lupus erythematosus, Alzheimer’s, atherosclerosis, macular degeneration, and endometriosis are associated with a chronic acute-phase response, oxidative stress, and dysfunctional HDL [25–30]. It is well documented that HDL is an important mediator of lipid homeostasis and provides multifaceted protection against oxidative stress and damage to vascular endothelium. Many of HDL’s beneficial functions in ameliorating inflammation and proliferation have been demonstrated in animal models [17–21]. HDL therapies using recombinant HDL (rHDL), apoA-I (main protein component of HDL), and apoA-I mimetic peptides are effective in animal models of lipid-mediated inflammatory diseases [31]. Our previous studies also showed that apoA-I mimetic peptides inhibit tumor development in mouse models of colon and ovarian cancer [17–21]. Moreover, epidemiological data have shown an inverse correlation between HDL-cholesterol (HDL-C) and cancer risk and suggested that HDL-C could potentially be used as a predictive measure for survival prognosis in certain types of cancer [32–33]. Despite the efforts, to date, HDL-based therapies have not yet been approved clinically. We exploited an inexpensive way to develop and test HDL therapy. Unlike in humans, HDL in cattle is abundant. We, therefore, tested whether bHDL can be an effective cancer therapy. We show that bHDL administered subcutaneously inhibited CT26 cell growth and CT26-mediated lung tumor development in BALB/C mice. We also demonstrated that ovarian tumor burden following ID8 cell injection was also significantly decreased in mice that received bHDL in chow. Our results demonstrate (Figure 1), for the first time, that bHDL can be an effective therapy for treating colon and ovarian cancers in mouse models.

Some of the HDL associated proteins have been recognized to confer, in part, the beneficial properties assigned to HDL. Among these, apoA-I, apoE, and apoJ (discussed below) have been studied extensively for their ability to contribute to the anti-inflammatory properties of HDL [17–21].

For example, apoA-I is the major protein constituent of HDL. Not surprisingly, concentrations of HDL and apoA-I were both found to be inversely associated with the risk of CC [15], NSCLC [27], and EOC [34]. A decreased level of pretherapy apoA-I was associated with worse survival in patients with NSCLC, and ovarian cancer. Serum apoA-I measurement before initial treatment may be a novel and routine biomarker to evaluate for metastasis and predict prognosis for NSCLC and ovarian cancer patients in daily clinical practice [35]. Most recently, Marinho et al reported that both apoA-I and apoA-I mimetic peptide were able to decrease the viability of EOC and CC cancer cell lines, SKOV3, CAOV3, and OVCAR3, *in vitro* [12]. The treatment with increasing concentrations of the peptide sensitized SKOV3 OVCAR3 and CAOV3 cells to cisplatin, a standard cytotoxic chemotherapeutic agent used to treat advanced EOC and CC [12]. This synergistic effect was observed both *in vitro* and *in vivo*, supporting an important role of apoA-I and apoA-I mimetic peptides as suppressors of ovarian tumorigenesis and as chemotherapy sensitizing agents.

Although ApoE and its specific isoforms have long been known to play a key role in lipid transport and atherosclerosis, the role of apoE in human cancers is not well understood. ApoE is differentially expressed in EOC versus serous borderline tumors and normal ovarian surface epithelium, implicating apoE as a potential tumor-associated marker in EOC [34]. Expression of apoE is significantly associated with a better 5-year survival outcome in patients who presented with peritoneal effusions at the time of diagnosis, inferring a protective role of apoE in the survival of EOC [34]. Cholesterol is recognized as a risk factor of aggressive prostate tumors, and dysfunctional ApoE2/E4 iso-form as a biomarker of aggressive disease [36] and ApoE containing HDL has been shown to overcome cholesterol-loaded adverse effects of HDL [37].

Heat shock proteins (HSPs) are stress-responsive molecules known to be crucial in many cancer types including ovarian cancer. ApoJ, a unique chaperone protein with analogous oncogenic criteria to HSPs, has been implicated in the diagnosis, prognosis, metastasis, and aggressiveness of various cancers, and plays a role in the pathogenesis of ovarian cancer [38]. ApoJ, a conserved glycoprotein that has been characterized from almost all human tissues and fluids, plays a key role in cellular stress response and survival. In humans, apoJ (Clusterin) is involved in many diseases related to oxidative stress, including atherosclerosis, neurodegenerative diseases, cancers, inflammatory diseases, and aging [39–42]. We have demonstrated that apoJ mimetic peptide renders HDL anti-inflammatory in mice and monkeys and dramatically reduces atherosclerosis in apolipoprotein E-null mice [25].

Several laboratories have developed peptide mimetics of HDL associated proteins with the intent to develop novel therapeutic strategies to treat inflammatory diseases. More recently, the field of dual-domain peptides has been pioneered by Dr. Anantharamaiah by combining apoA-I and apoE mimetic peptides into a single dual-domain peptide, AEM-28 [43]. Peptide AEM-28 attenuates the effects of oxidative stress on ApoE secretion, inhibits amyloid plaque deposition, and thus could be beneficial in the treatment of Alzheimer’s disease [30]. AEM-28 was compared with the well-studied anti-atherogenic apoA-I mimetic peptide 4F for reducing lesion formation in female apoE null mice with already existing lesions. Although both peptides had similar anti-inflammatory properties, AEM-28 was more effective in inhibiting lesions than 4F at the same dose, frequency, and route of administration, perhaps due to its cholesterol-reducing and anti-inflammatory properties [43, 44]. New acyl-analogues of AEM-28 have been shown to exhibit enhanced potency at lower doses than AEM-28 in dyslipidemic mouse and monkey models which may make them attractive therapeutic candidates for clinical trials [25, 45]. Most recently, AEM-28–2 has been reported to attenuate cellular injury in LPS-treated THP-1 macrophages, facilitate the removal of cellular debris and damaged organelles via induction of autophagy and modulate apoptosis in cancer cell lines [46].

Our results demonstrated, for the first time, that peptides AEM-28 and AEM-28–2 significantly decrease tumor development following CT26 cell injection. AEM-28 and AEM-28–2 inhibited the viability of chemotherapy-resistant human cancer cell lines, OV2008 and SKOV3, and reduced cell viability and induced apoptosis in CT26 cells *in vitro*. Recently, AEM-28 and AEM-28–2 have been confirmed to possess significant anti-inflammatory properties, issued orphan drug status by the FDA, and entered clinical trials to assess the efficacy of atherogenic lipid clearance from the circulation [25, 45]. In our studies, AEM-28–2 was clearly more superior than AEM-28 in preventing the development and growth of tumors in both colon and ovarian cancer models. This may be due to the better LDL clearance of AEM-28–2.

We also show that the novel 20 amino acid residue peptide, HM-10/10 potently inhibits tumor development and growth in both colon and ovarian cancer models. We have recently reported that HM-10/10 protects against chemically-induced macular degeneration in mice [47]. The apoE mimetic portion of AEM-28–2 and HM-10/10 are the same while the anti-inflammatory parts are different; an 18 amino acid apoA-I mimetic in the case of AEM-28–2 and a 10 amino acid apoJ mimetic in HM-10/10. Future studies will determine which of these two potent anti-tumorigenic dual-domain peptides will be efficacious in higher organisms.

LPA, a platelet-activating component of mildly oxidized LDL, plays an important role in vascular biology and ovarian cancer [48, 49]. Our results demonstrate that bHDL and dual-domain peptides HM-10/10 and AEM-28–2 significantly reduce plasma levels of LPA in mice warranting more studies on the mechanisms by which LPA modulates tumor development and growth. In summary, we report for the first time that bHDL and novel dual-domain HDL mimetic peptides exert potent anticancer effects both *in vitro* and *in vivo* representing new and novel targeted therapies to treat devastating chemotherapy-resistant EOC and CC. While bHDL may not be therapeutically used due to antibody production, clinical trials will be required to assess the efficacy of dual-domain peptides as new promising pharmaceutical agents.

## Abbreviation:

HDL: high-density lipoprotein
LDL: low-density lipoprotein
apoA-I: apolipoprotein A-I
apoJ: apolipoprotein J
apoE: apolipoprotein E
LPA: lysophosphatidic acid
